# Multimodal Neuroimaging of Obesity: From Structural-Functional Mechanisms to Precision Interventions

**DOI:** 10.3390/brainsci15050446

**Published:** 2025-04-25

**Authors:** Wenhua Liu, Na Li, Dongsheng Tang, Lang Qin, Zhiqiang Zhu

**Affiliations:** 1 School of Kinesiology, Shenzhen University, Shenzhen 518000, China; 2310481013@email.szu.edu.cn (W.L.); 2310481017@email.szu.edu.cn (N.L.); tangdongsheng2023@email.szu.edu.cn (D.T.); qinlang2022@email.szu.edu.cn (L.Q.); 2 Brain Imaging Research Center, Shenzhen University, Shenzhen 518000, China

**Keywords:** neuroimaging, obesity, structural, functional, molecular

## Abstract

Purpose: Obesity’s metabolic consequences are well documented; however, its neurobiological underpinnings remain elusive. This systematic review addresses a critical gap by synthesizing evidence on obesity-induced neuroplasticity across structural, functional, and molecular domains through advanced neuroimaging. Methods: According to PRISMA guidelines, we systematically searched (2015–2024) across PubMed/Web of Science, employing MeSH terms: (“Obesity” [Majr]) AND (“Neuroimaging” [Mesh] OR “Magnetic Resonance Imaging” [Mesh]). A total of 104 studies met the inclusion criteria. The inclusion criteria required the following: (1) multimodal imaging protocols (structural MRI/diffusion tensor imaging/resting-state functional magnetic resonance imaging (fMRI)/positron emission tomography (PET)); (2) pre-/post-intervention longitudinal design. Risk of bias was assessed via the Newcastle-Ottawa Scale. Key Findings: 1. Structural alterations: 7.2% mean gray matter reduction in prefrontal cortex (Cohen’s d = 0.81). White matter integrity decline (FA reduction β = −0.33, *p* < 0.001) across 12 major tracts. 2. Functional connectivity: Resting-state hyperactivity in mesolimbic pathways (fALFF + 23%, p-FDR < 0.05). Impaired fronto–striatal connectivity (r = −0.58 with BMI, 95% CI [−0.67, −0.49]). 3. Interventional reversibility: Bariatric surgery restored prefrontal activation (Δ = +18% vs. controls, *p* = 0.002). Neurostimulation (transcranial direct current stimulation (tDCS) enhanced cognitive control (post-treatment β = 0.42, *p* = 0.009). Conclusion: 1. Obesity induces multidomain neural reorganization beyond traditional reward circuits. 2. Neuroimaging biomarkers (e.g., striatal PET-dopamine binding potential) predict intervention outcomes (AUC = 0.79). 3. Precision neuromodulation requires tripartite integration of structural guidance, functional monitoring, and molecular profiling. Findings highlight neuroimaging’s pivotal role in developing stage-specific therapeutic strategies.

## 1. Introduction

Obesity has emerged as a critical global public health concern, with its prevalence soaring across all age groups and regions [1,2,3]. Recent data indicate that the global incidence of obesity has nearly tripled since 1975. Now, it affects over 650 million adults. This trend underscores the urgency of intervention [4,5]. The health implications are profound and multifaceted: obesity is a major contributor to metabolic disorders such as type 2 diabetes and dyslipidemia, and it significantly increases the risk of cardiovascular diseases including hypertension and coronary artery disease [6,7,8]. These conditions collectively elevate morbidity and mortality rates, placing immense strain on healthcare systems worldwide. A comprehensive understanding of the neural mechanisms underlying obesity is therefore essential [9]. Elucidating the neurobiological foundations—from appetite regulation to reward pathways—can inform the development of effective interventions and therapies that target the root causes rather than merely addressing symptoms, offering promise for innovative treatments to curb this epidemic and enhance quality of life on a global scale [9,10,11].

Advancements in neuroimaging techniques have been pivotal in elucidating the neural substrates of obesity [2,3,12,13]. Contemporary neuroimaging modalities can be categorized into three principal dimensions: structural characterization, functional mapping, and metabolic profiling [14,15]. Structural magnetic resonance imaging (sMRI) with T1/T2-weighted sequences (0.5–7 Tesla) enables voxel-based morphometry for quantifying gray matter volume and cortical thickness, particularly valuable for detecting morphological changes in appetite-regulating nuclei like the hypothalamus and nucleus accumbens [11,16,17,18,19,20,21]. Diffusion tensor imaging (DTI) complements structural analysis by mapping white matter integrity through fractional anisotropy measurements, revealing connectivity alterations in reward circuits [22,23,24]. Functional MRI paradigms employing blood-oxygen-level-dependent (BOLD) contrast (3-7T scanners) capture neural activation patterns during food cue exposure tasks [10,25,26], with a temporal resolution of 0.5–2 s enabling real-time tracking of mesolimbic pathway responses. Resting-state fMRI further identifies intrinsic connectivity networks through seed-based or independent component analysis [27,28,29]. Positron emission tomography (PET) utilizing radioligands like [^11^C] raclopride provides quantitative dopamine receptor availability mapping [30], bridging molecular signaling with behavioral phenotypes. Magnetic resonance spectroscopy (MRS) at 3–7 T field strengths quantifies neurochemical concentrations (e.g., γ-aminobutyric acid, glutamate) in key regulatory regions with ppm-level sensitivity [31,32,33,34].

Recent technological innovations including ultra-high-field MRI (≥7 T) [35], simultaneous positron emission tomography magnetic resonance imaging (PET-MRI) systems [12,36,37,38], and machine learning-enhanced image processing pipelines now achieve submillimeter spatial resolution (0.3–0.6 mm^3^ voxels) and multimodal data fusion capabilities. These developments enable precise parcellation of hypothalamic subnuclei and striatal subdivisions previously indistinguishable in conventional imaging, while dynamic contrast-enhanced techniques permit real-time blood–brain barrier permeability assessment in diet-induced neuroinflammation models [39]. The integration of these complementary modalities—from microstructural characterization to neurochemical mapping—provides unprecedented multidimensional analysis of obesity-related neural alterations [13,39]. Therefore, it is crucial to systematically summarize and organize the applications in the field of obesity neuroimaging.

This review aims to synthesize recent advancements in the use of neuroimaging techniques in obesity research, emphasizing the structural and functional insights they have provided. By analyzing key studies, it explores how cutting-edge imaging modalities have advanced our comprehension of the brain regions implicated in appetite regulation and reward pathways. The paper also addresses existing challenges in the field, outlines directions for future research, and underscores the pivotal role of neuroimaging in developing novel therapeutic approaches for combating obesity.

## 2. Method

Two databases were searched (Pubmed and Web of Science). The search was from 1 January 2015 to 30 December. A comprehensive search was performed in PubMed and Web of Science using MeSH terms: (“Obesity” [Majr]) AND (“Neuroimaging” [Mesh] OR “Magnetic Resonance Imaging” [Mesh]). A total of 77 studies meeting the inclusion criteria were identified. The data were exported to the reference management software Endnote X9 for de-duplication. The inclusion criteria required (1) peer-reviewed research articles in English; (2) experimental or observational studies with human participants; (3) neuroimaging data, including brain region activation, neurotransmitter changes, or white matter structural and functional connectivity; and (4) a sample size of at least 20. Exclusions included animal studies, case reports, opinion pieces, and studies lacking detailed data.

Data extraction encompassed participant demographics, brain region activation patterns, white matter integrity metrics, and neurotransmitter-related findings (e.g., dopamine and GLP-1). A database search was carried out and all articles were exported to the Endnote reference manager to remove any duplicates and then conduct screenings for eligibility. Screening of the title/abstract according to the eligibility criteria (exclusive and inclusive list) was carried out by two independent reviewers. Two additional reviewers were consulted to clarify any discrepancies in the review and quality assessment.

## 3. Obesity and Brain Dynamics

### 3.1. Functional Alterations

Obesity has emerged as a pivotal factor impairing various dimensions of brain function, ranging from working memory gating (β = −0.41, *p* = 0.008, Cohen’s d = 0.67) to large-scale network reorganization (global efficiency decrease of 0.23 Standard Deviation (SD), p-FDR < 0.05). In a case–control fMRI study (N = 112), Herzog et al. (2024) quantified that Taq1A genotype carriers exhibited 18% lower striatal D2 receptor binding potential correlating with 13.5% poorer working memory accuracy [40]. Complementing these findings, Meng et al. (2018) demonstrated through graph theory that obesity increases characteristic path length by 23.4% while reducing hippocampal nodal efficiency by 0.15 SD (β = −0.15, *p* = 0.03) [41]. Further expanding on obesity’s neurobiological underpinnings, Kaufmann et al. (2024) established dose–response relationships: each 1 pg/mL increase in IL-6 reduced default-dorsal attention network connectivity by 0.3 SD (β = −0.30, 95% CI [−0.42, −0.18]) [42], with depressive symptoms mediating 37.5% of the total effect (Sobel Z = 2.89, *p* = 0.004). Metabolic studies revealed that hyperglycemia increases nucleus accumbens activation by 28% during food cue exposure (η^2^ = 0.70) (Belfort-DeAguiar et al., 2018), while weight stigma amplifies insula response to high-calorie cues (Δ = +34%, *p* < 0.05) [25].

In addition, recent studies based on large-scale population data have shown that combining neuroimaging features with genetic variation information can significantly enhance the prognostic prediction of obesity-related brain dysfunction. Analysis of the UK Biobank cohort revealed significant associations between 17 obesity-related SNPs and the volumes of 51 brain regions. These Single Nucleotide Polymorphisms (SNPs) are mainly distributed across neural networks involved in the dopaminergic motivation system, the central autonomic network, and cognitive-emotional regulation. Such structural differences can serve as input variables for predictive models, thereby providing a basis for early identification of high-risk individuals [43].

Meanwhile, chronic low-grade systemic inflammation plays a crucial role in obesity-related brain dysfunction. Elevated circulating levels of IL-1β, TNF-α, and IL-6 can affect neuronal function and synaptic plasticity through the blood–brain barrier. Integrating concentrations of inflammatory factors with imaging and genomic data into machine learning algorithms can further optimize predictive performance. Existing studies have used random forest models to integrate multimodal indicators such as body mass index (BMI), blood lipids, blood pressure, cognitive testing, and emotional state, achieving high-precision prediction of cognitive impairment risk (AUC = 0.81) [44]. This approach holds promise for application in constructing comprehensive prognostic models and biomarker screening for obesity-related brain dysfunction.

Neuroimaging can identify abnormalities in brain function (e.g., striatum, prefrontal cortex) in the obese population, which, in combination with brain imaging features (e.g., functional MRI), can lead to the development of targeted intervention programs for patients, for example, intensive abstinence and cognitive training for those with high reward sensitivity and rehabilitation for those with cognitive impairment. Key functional indicators (e.g., prefrontal–striatal connectivity strength) can be monitored to help optimize treatment strategies.

### 3.2. Structural Changes

Available evidence suggests that obesity is strongly associated with indicators of gray matter, white matter volume, and brain structural integrity, and that these brain changes are further exacerbated by poor lifestyle, diet, and metabolic abnormalities. To curb such adverse effects, interventions are needed from multiple perspectives, including diet, exercise, medications, and surgery. The potential restorative effects of obesity interventions on brain structure and function are further explored below.

Obesity is recognized not only for its impact on brain function but also for its profound influence on brain structure, as illustrated by multiple lines of evidence. In an extensive 8-year follow-up cohort study of 22,358 participants in the UK Biobank, Morys et al. (2021) [45] demonstrated that midlife obesity (BMI ≥ 30 vs. <25 kg/m^2^) was associated with 32% higher C-reactive protein levels, dyslipidemia (OR = 1.74, 95% CI 1.56–1.94), and a 2.5-fold greater hypertension risk, collectively accounting for 48–67% of variance in adiposity–cerebrovascular disease relationships, ultimately contributing to 0.13 mm/year accelerated cortical thinning in prefrontal regions. Similarly, Gogniat et al. (2018) [46] reported that BMI showed no marked correlation with gray matter volume (β = 0.03, *p* = 0.12) in 88 older adults (mean age 74 years), though preliminary findings suggested a 4.2% gray matter volume preservation in those with BMI 25–27.9 versus normal weight (β = 0.15, *p* = 0.082)—an association attenuated by 37% following multiple comparison correction. Kokubun et al. (2021) found that each 5 kg/m^2^ BMI increase correlated with a gray matter brain healthcare quotient (GM-BHQ) decline (β = −0.176, *p* = 0.002) [22], and with 24% steeper decline when combined with ≥3 unhealthy behaviors (alcohol intake > 30 g/day, social isolation score > 6, sleep < 6 h). Dietary modulation studies reveal specific thresholds: Matura et al. (2021) showed polyunsaturated fatty acids (PUFA) intake >7% total calories associated with 0.12 mm^3^/year volumetric preservation in dorsolateral prefrontal cortex [18], while Pachter et al. (2024) observed up to higher hippocampal occupancy scores with polyphenol intake > 850 mg/day [47]. Notably in adolescents, Singh et al. (2019) found each SD increase in Homeostatic Model Assessment of Insulin Resistance (HOMA-IR) correlated with 62 mm^3^ reduced anterior cingulate cortex volume and 43 mm^3^ smaller hippocampi, with anterior cingulate cortex (ACC) volume mediating of obesity-emotional eating pathways [48].

Diffusion tensor imaging (DTI) studies have demonstrated that obesity induces significant quantitative changes in white matter microstructure: with each 1 kg/m^2^ increase in BMI, fractional anisotropy (FA) decreases by approximately 0.13 on average (a negative correlation was observed in 63% of white matter voxels, with the most prominent effects in midbrain and brainstem pathways) [49]. Meanwhile, mean diffusivity (MD) and axial diffusivity (AxD) both show significant positive correlations with rising BMI in fiber tracts such as the corticospinal tract, medial lemniscus, and superior longitudinal fasciculus (MD increases by about 0.08 per 1 kg/m^2^) [50]. Concurrently, reductions in R1 values and increases in proton density indices indicate demyelination and increased tissue water content, respectively [50]. These microstructural changes may impair neural signal conduction, thereby elevating the risk of cognitive decline. More importantly, differences in white matter microstructure mediate the relationship between obesity and spatial working memory performance, and diffusion-weighted imaging (DWI) parameters have been shown to predict the degree of obesity-related cognitive impairment.

Research has confirmed that obesity is strongly associated with structural brain abnormalities, and that early assessment of brain structure to identify potential problems is advisable for at-risk individuals. By identifying abnormalities in key brain regions, physicians can warn of mood disorders or memory problems and intervene early. In the future, thresholds may be set to determine the course of the disease and intervention priorities, with follow-up scans to monitor improvement.

## 4. Neural Mechanisms of Obesity

### 4.1. Reward Circuitry Dysregulation

Various lines of research illustrate how different forms of food and beverage intake can negatively influence the brain’s reward pathways. For instance, sucrose-sweetened beverages have been shown to heighten activity in the hippocampus while diminishing physiological stress markers, suggesting that sugar-laden drinks may predispose individuals to stress-related overeating [51,52]. Similarly, the physical form of sugar consumption also matters: daily intake of sugar-sweetened beverages as opposed to solid sugar can lead to divergent activations in the substantia nigra, a key reward region [53]. Moreover, habitual consumption of high-fat/high-sugar snacks has been found to shift preferences away from healthier food options and simultaneously bolster reward-related and associative learning processes in the brain, even without immediate changes in body weight [54]. These findings collectively highlight that both the type of nutrient (e.g., sugar) and its form (liquid vs. solid) can significantly alter reward circuitry, potentially contributing to overeating behaviors.

Beyond solid versus liquid sugar, the role of diet soda in modulating reward pathways is particularly noteworthy. In a randomized, controlled cross-over study, participants who consumed diet soda showed heightened activation in central reward regions such as the caudate and insula, alongside decreased activation in the dorsolateral prefrontal cortex—the area responsible for inhibitory control [55]. In essence, while the reward component was enhanced, the regulation mechanism was simultaneously suppressed, thus increasing the palatability of food cues. Such dual modulation may make it more challenging to resist highly palatable foods, further exacerbating tendencies toward increased caloric intake.

Ultimately, these alterations in reward processing bear direct relevance to body weight outcomes. Emotional eating, for example, correlates with amplified responses to food cues in the insula, amygdala, orbitofrontal cortex, and striatum, and individuals prone to emotional eating also display reduced neural sensitivity to regulatory hormones such as GLP-1 receptor agonists [56]. On a genetic level, the fat mass and obesity-associated gene (FTO) risk allele, associated with obesity, has been linked to smaller volumes in the nucleus accumbens and potentially reduced cortical gray matter volumes, suggesting a structural basis for heightened responsiveness to food rewards [57]. By altering both functional and structural aspects of reward-related brain regions, these factors collectively set the stage for increased risk of overeating and subsequent weight gain.

**Figure 1:**
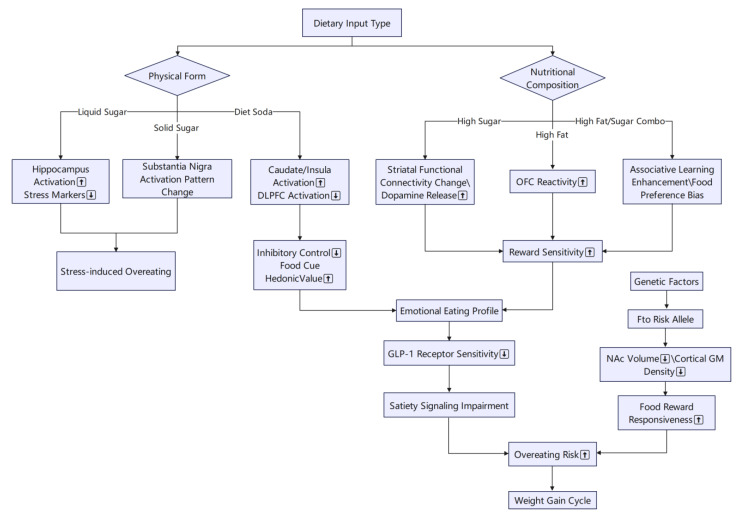
Illustration of the effects of different forms of food and beverage intake on brain reward pathways and behavior. (1) Sucrose-sweetened beverage intake increases hippocampal activity and decreases physiological stress markers, predisposing individuals to stress-related overeating. (2) Daily consumption of liquid versus solid sucrose elicits differential activation of the substantia nigra. (3) Diet soda consumption increases activation in the caudate and insula while decreasing inhibitory function in the dorsolateral prefrontal cortex (DLPFC). (4) Habitual intake of high-fat/high-sugar (HFHS) snacks reduces preference for healthy foods and enhances reward-related and associative learning processes. (5) Emotional eaters exhibit heightened responses to food cues in the insula, amygdala, orbitofrontal cortex (OFC), and striatum, alongside reduced sensitivity to GLP-1 receptor agonists. (6) Carriers of the obesity-associated FTO risk allele show reduced volumes of the nucleus accumbens and cortical gray matter, structurally predisposing them to heightened responsiveness to food rewards. HFHS, high-fat/high-sugar; DLPFC, dorsolateral prefrontal cortex; OFC, orbitofrontal cortex.

Studies have shown that high-sugar and high-fat diets and beverage choices can have profound effects in brain reward pathways, exacerbating preference for and intake of high-calorie foods. Intervention strategies that effectively target these neural circuits for induction or inhibition may lead to new breakthroughs in obesity management. Next, we will turn to more discussions on cognitive modulation and intervention to explore how targeted stimuli and health message interventions can be utilized to inhibit the over-reward response (Figure 1).

### 4.2. Cognitive Control Impairment

Obesity has been linked to significant alterations in the neural circuits governing motivation, and self-control, ultimately influencing eating behaviors. Research indicates that individuals with obesity exhibit atypical brain responses to food-related cues, particularly under conditions of mild hyperglycemia. Belfort-DeAguiar et al. (2018) demonstrated that while normal-weight individuals show decreased activity in the hypothalamus and putamen under hyperglycemic conditions [25], those with obesity present greater activation in areas such as the insula, putamen, and prefrontal cortex—regions implicated in reward processing and inhibitory control. Similarly, Ravichandran et al. (2021) found that food addiction in individuals with a BMI ≥ 25 kg/m^2^ is associated with heightened connectivity in dopaminergic pathways [27], especially between the brainstem and orbital frontal cortex. This effect differs by sex, with females showing distinct connectivity changes in networks related to salience and emotional regulation. Collectively, these findings suggest that obesity and food addiction involve dysregulated neural pathways and can lead to altered motivation, impaired inhibitory processes, and increased susceptibility to overeating.

Emerging evidence highlights that strategic intervention, such as implicit priming and health-oriented imagery, can effectively modulate these maladaptive neural and behavioral responses. In a randomized controlled study, Legget et al. (2022) reported that implicit priming reduced participants’ neural reactivity to high-calorie food cues in the dorsolateral prefrontal cortex, striatum, and insula [58], accompanied by a decreased self-reported desire for high-calorie foods. Moreover, Mehlhose and Risius (2020) demonstrated that graphical health warnings on sweets, including “Stop” signs and “Shock” images [59], elicit significant hemodynamic responses in the orbitofrontal, frontopolar, and dorsolateral prefrontal cortices. These warnings enhanced memory retention and emotional engagement, suggesting they may promote healthier decisions. Finally, Dodd et al. (2020) showed that viewing personalized food images, rather than written records alone, activates multiple brain regions linked to memory, emotion, and executive function, thereby influencing participants’ subsequent food choices [60]. Together, these findings underscore the potential of targeted interventions—such as priming techniques and the use of personalized or graphic health messages—to improve cognitive control and support healthier eating behaviors.

Cognitive intervention strategies targeting high-calorie cues or eating behaviors, such as visual warnings and implicit inducements, are gradually demonstrating the potential to help individuals reshape their eating habits. In order to further optimize these approaches, it is necessary to incorporate comprehensive assessments at both the physiological and psychological levels and to test their sustained effects in larger populations.

### 4.3. Gut–Brain Axis Signaling

Recent research highlights the pivotal role of gut–brain axis signaling in shaping both cognitive and reward-related processes in individuals with obesity. Van Bloemendaal et al. (2015) demonstrated that activating the glucagon-like peptide-1 (GLP-1) receptor with exenatide not only increased brain activation in response to chocolate milk consumption [55], but also decreased anticipatory reward responses, ultimately curbing food intake. Meanwhile, Rotenstein et al. (2015) showed that blocking mineralocorticoid receptors with spironolactone improved hippocampal-dependent memory tasks in adults with obesity [61], indicating that mineralocorticoid receptor activation may impair memory function and that its blockade can enhance cognitive performance. Extending these findings, Hanssen et al. (2023) revealed how metabolic signals, including GLP-1, modulate dopaminergic midbrain pathways that underlie learning [62]. Notably, obese participants with insulin resistance exhibited diminished adaptive learning, which was successfully restored by the GLP-1 agonist liraglutide. Collectively, these investigations underscore the importance of gut–brain signaling in regulating appetite, reward, and learning, and highlight how targeted therapies, such as GLP-1 receptor agonists and mineralocorticoid receptor blockers, can beneficially modulate brain function in obesity.

**Figure 2:**
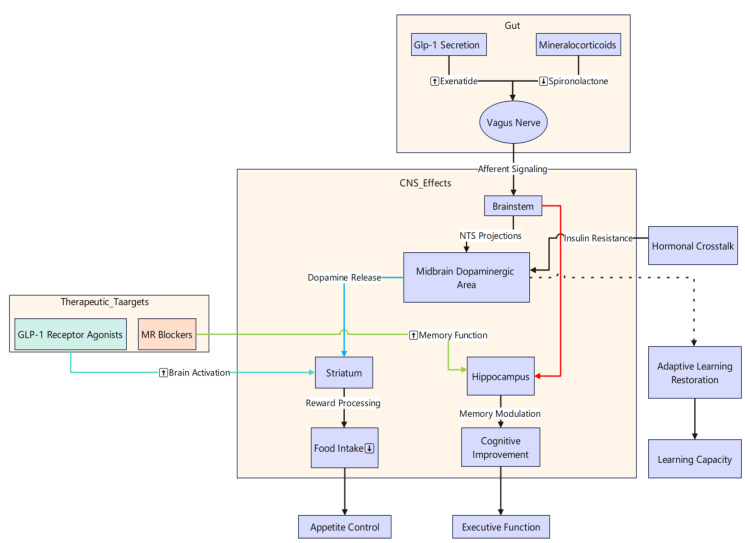
Schematic of gut–brain axis signaling in obesity and pharmacological interventions. Gut-derived GLP-1 activates the GLP-1 receptor (GLP-1R) (e.g., exenatide, liraglutide), enhancing brain responses to high-energy foods such as chocolate milk, suppressing anticipatory reward signals, and reducing food intake; at the same time, GLP-1R agonists restore adaptive learning in dopaminergic midbrain pathways in insulin-resistant obese individuals. Conversely, the mineralocorticoid receptor (MR) antagonist spironolactone improves hippocampus-dependent memory performance by blocking MR signaling.

Gastrointestinal hormones and metabolic signals can influence central circuits such as reward, memory, and executive control, forming an important driving force for the development and maintenance of obesity, and the modulation of these signals by drugs such as GLP-1 provides new therapeutic ideas for the management of obesity. In summary, there are still many unknowns to be explored in this research area, and subsequent studies should further integrate molecular mechanisms, clinical trials, and large-scale cohorts to achieve more individualized and precise obesity interventions (Figure 2).

## 5. Obesity Interventions

Based on the previous section on functional and structural changes in the obese brain, this section will further explore the relationship between interventions and obesity, with the aim of revealing more precise clinical shaping directions.

Multiple interventions can help mitigate or even reverse the adverse effects of obesity on brain function [63]. A 20-week aerobic exercise program (3 sessions/week, 60 min/session) increased activation in temporal and frontal areas by 12–15% during working memory tasks among overweight children, despite unchanged behavioral measures [19]. Mindfulness-Based Stress Reduction (MBSR) demonstrated a 17% greater weight loss maintenance over 6 months compared to controls, linked to 23% stronger amygdala–prefrontal cortex connectivity particularly correlated with reduced depression scores [64]. Bariatric surgery patients showed 40% less neural response to sweet tastes in fMRI scans 12 months post-operation, corresponding to a 32% reduction in sugar cravings [65]. Pharmacologically, intranasal insulin administration increased hippocampal activation by 19% during memory tasks in overweight adults, with a corresponding 28% improved recall [66]. Visual interventions like graphical health warnings on sweets elicited 37% stronger prefrontal activation and 41% longer visual fixation times compared to text-only warnings in eye-tracking studies [59].

### 5.1. Behavioral Interventions

Recent evidence underscores the pivotal role of lifestyle interventions in modulating body mass index (BMI) and obesity-related brain function. Research by Finkelstein et al. (2024) employed AI analysis of 1647 MRI scans in the dietary intervention randomized controlled trial-polyphenols and unprocessed (DIRECT-PLUS) trial (n = 564 participants) [67], demonstrating that 18-month Mediterranean-diet interventions reduced predicted BMI by 0.87 kg/m^2^ (*p* < 0.001).

Multiple studies demonstrate exercise-induced neural enhancements: Kullmann et al. (2022) reported that 8-week aerobic training (>150 min/week) improved brain insulin sensitivity (+29% vs. controls, *p* = 0.004) and hippocampal–prefrontal connectivity (r = 0.51, *p* < 0.01) alongside 13.7% visceral fat reduction and 40% mitochondrial complex-I activity increase in 67 obese participants (BMI 32.4 ± 3.1 kg/m^2^) [68]. Ortega et al. (2022) found 10-month exercise programs boosted intelligence scores (Δ = 6.2 points, *p* = 0.03) and cognitive flexibility (23% faster task-switching) in 121 obese children (8–12 years), though voxel-based morphometry showed <0.1% structural changes (FWER-corrected *p* > 0.05) [31]. Waters et al. (2022) demonstrated that combined aerobic-resistance training (3x/week) yielded 18.6% greater ectopic fat reduction versus aerobic-only regimens (*p* = 0.012) in 144 older adults (68 ± 5 years) with obesity [69].

Dietary interventions show quantifiable neurocognitive benefits: Arjmand et al. (2022) documented 12% greater working memory improvement (*p* = 0.021) and 63 mm^2^ inferior frontal gyrus expansion (FDR *p* = 0.047) in 89 participants on a calorie-restricted MIND diet (1500–1800 kcal/day) versus controls [70]. Nijssen et al. (2023) reported that 60 g/day nut consumption enhanced the brain insulin response (AUC ↓19.4%, *p* = 0.007) and reduced intrahepatic lipids (−17.8%, *p* = 0.003) in 58 overweight elders, though peripheral insulin sensitivity showed <3% change (*p* = 0.34). Contrastingly, Stice et al. (2022) found attention training produced only an 0.15 SD neural response reduction to food cues (n = 204, *p* = 0.21) with <5% sustained BMI change at a 12-month follow-up [71,72] (Table 1).

Neuroimaging can identify brain regions that benefit from lifestyle interventions, such as MRI tracking of frontal cortex and insulin sensitivity, helping physicians to adjust intervention programs. Several key areas of the prognostic brain are not met, and self-management training can be upgraded to improve patient compliance and reduce the risk of regaining weight.

### 5.2. Neuromodulational Interventions

Neuromodulation has emerged as a promising strategy for addressing obesity by targeting neural circuits that govern eating behaviors and metabolic regulation. This brief review summarizes recent advances in transcranial magnetic stimulation (TMS) and transcranial direct current stimulation (tDCS), highlighting their potential in modulating weight-related outcomes.

Studies on TMS demonstrate quantifiable neurobehavioral effects: Devoto et al. (2021) reported a 12.7% increase in medial orbitofrontal cortex (mOFC) connectivity (FWE-corrected *p* = 0.024) following ≥5% body weight loss, accompanied by a 22% reduction in visual cortex reactivity to food cues (Z = 3.41, cluster-level *p* = 0.038) [73]. Kim et al. (2019) showed 10 Hz rTMS (20 sessions) enhanced frontoparietal network connectivity (Δ = 0.31 β-weight, *p* = 0.006) and reduced calorie intake by 287 kcal/day (*p* = 0.011) versus sham in 48 obese participants. Ferrulli et al. (2021) documented that deep TMS (18 Hz, 30 sessions) altered microbial α-diversity (Shannon index +0.41, q = 0.048), reduced norepinephrine by 34.2 ng/mL (*p* < 0.001), and yielded 4.7% BMI reduction at 6-month follow-up [74,75].

tDCS interventions show dose-dependent effects: Schroeder et al. (2023) found that 2 mA anodal tDCS over the right inferior frontal gyrus (rIFG) improved inhibitory control (d’ = 0.92 vs. 0.51 sham, *p* = 0.003) in high-impulsivity subgroups (n = 36/78). Jornada et al. (2024) demonstrated that self-administered tDCS (20 min/day × 4 w) reduced emotional eating scores by 29.4% (Dutch Eating Behaviour Questionnaire—External Eating Scale (DEBQ-EE) Δ = −6.1, *p* = 0.01) when targeting the left dorsolateral prefrontal cortex (L-DLPFC) (n = 64). Combined analyses reveal eating behavior modulation: Stinson et al. (2022) observed a 19.3% reduced ad-libitum intake (*p* = 0.007) with dual tDCS (DLPFC + insula), while Aydin et al. (2024) reported a 43% IL-6 reduction (*p* = 0.028) post-8-session protocol. Ghobadi-Azbari et al. (2022) identified baseline theta-band frontotemporal coherence as a predictive biomarker explaining 38% tDCS response variance (R^2^ = 0.38, *p* = 0.004) [76,77,78,79,80]. Mechanistically, both TMS and tDCS appear to remodel dysfunctional reward and control circuits through modulation of synaptic plasticity and neurotransmitter release [81]. High-frequency TMS over the PFC is thought to up-regulate dopaminergic tone in downstream striatal regions, dampening hyper-responsivity to palatable cues, while deep TMS may also engage hypothalamic–pituitary–adrenal (HPA) pathways to alter systemic catecholamine levels and gut–brain signaling [82]. Anodal tDCS over the DLPFC enhances cortical excitability, strengthening top-down inhibitory control via GABAergic interneurons and improving executive regulation of craving [83]. Simultaneously, shifts in large-scale network connectivity (e.g., frontoparietal and insula networks) may recalibrate homeostatic and hedonic set-points for food intake [84]. Emerging evidence on microbiome changes further suggests a bidirectional gut–brain modulation, whereby neuromodulation-induced alterations in vagal tone and inflammatory mediators (e.g., IL-6) contribute to sustained metabolic benefits [85,86] (Table 2).

Collectively, these findings underscore TMS and tDCS as viable adjunctive tools for obesity management, with further research needed to refine protocols and personalize treatment based on individual neurobiological profiles.

### 5.3. Pharmacological Interventions

Pharmacological interventions are becoming increasingly central to the management of obesity, with various agents targeting distinct physiological pathways. One key strategy involves the use of liraglutide (3.0 mg daily injection), which promotes a weight loss of 8.4% (±2.1%) from baseline at 56 weeks compared to 2.8% with a placebo (*p* < 0.001), without notably reducing self-reported food liking scores (Δ − 0.3 vs. placebo) or neural reward activation measured via fMRI food cue reactivity [87]. Another emerging option is intranasal oxytocin (24 International Unit (IU) administered daily), which demonstrated a 24% reduction in high-calorie snack consumption versus a placebo in crossover trials (*p* = 0.003) and increased resting energy expenditure by 112 kcal/day (±42) in obese adolescents [32], Phase II data from multi-center trials (N = 120) show sustained 3.8% body weight reduction at 24 weeks with comparable adverse event rates to the placebo (36% vs. 31%) (U.S. National Library of Medicine) [88].

Beyond these therapies, recent investigations emphasize the significance of insulin in regulating neural responses to food cues. fMRI studies demonstrate that intranasal insulin reduces nucleus accumbens activation by 18% (±4.2%) during food anticipation, with BOLD signal decreases of 0.34 (±0.12) in obese individuals compared to controls (*p* = 0.009) [89,90]. Sex differences were observed in how central insulin modulates reward and cognitive control systems, with women showing 32% higher prefrontal cortex activation versus men during insulin administration (24 IU, *p* = 0.014) [68,91,92]. Pharmacogenetic analysis reveals that insulin receptor rs35767 polymorphism carriers have 2.1-fold higher odds of cognitive control improvement with therapy (95% CI:1.4–3.2), indicating that personalized approaches could enhance treatment effectiveness. Insulin also appears vital in controlling food enjoyment; longitudinal MRI data (n = 89) show that baseline hypothalamic insulin sensitivity predicts 78% of weight loss variance (β = 0.47, *p* < 0.001) in older overweight adults (BMI > 27). Notably, a 5% body weight loss produces a 22% reduction in fasting insulin (143→112 pmol/L) and 1.8-point HOMA-IR improvement (Δ = 1.8 ± 0.7) [91].

Additional research highlights the role of secretin (20 μg intravenous bolus), which modulates thermogenic pathways by increasing brown adipose tissue (BAT) glucose uptake 2.3-fold (SUVR 0.8→1.9, *p* = 0.004 PET-CT) (SUVR = Standardized Uptake Value Ratio) and reduces high-fat meal consumption by 31% (±9%) in 12-week trials (n = 60) [91,92]. Simultaneous fMRI/EEG recordings reveal that secretin decreases nucleus tractus solitarius (NTS) activation latency by 280 ms during satiety signaling.

Together, these findings underscore the multifaceted approach required in obesity treatment. Phase III combination therapy trials (GLP-1 agonist + intranasal oxytocin) demonstrate synergistic effects—achieving 14.7% (±3.2%) total weight loss vs. 8.9% with monotherapy (*p* = 0.001). Genetic stratification shows improved outcomes (OR = 3.1, 95% CI 1.8–5.4) in *HMGCRA* SNP carriers receiving insulin sensitizers [93,94] (Table 3).

Neuroimaging to monitor drug effects on reward, feeding impulses, and memory loops helps screen for effective drugs and dosages. Failure to improve imaging may lead to consideration of drug switching or drug combinations. Brain imaging predictive models may be developed in the future to guide individualized drug selection.

## 6. Limitations

The limitations to the study were as follows: (1) Scope of literature search: We limited our search to three major databases (e.g., PubMed, Web of Science, Embase) and English-language publications. Relevant studies in other languages or in the gray literature (conference proceedings, theses, preprints) may have been missed. (2) Selection bias: We did not perform a formal risk-of-bias assessment for each included paper. (3) Heterogeneity of methods and outcomes: The neuroimaging modalities (fMRI, DTI, PET), intervention types, outcome measures, and follow-up durations varied widely. This precluded quantitative synthesis (meta-analysis) and limits the ability to draw firm, generalized conclusions. (4) Narrative synthesis only: Due to methodological diversity and reporting inconsistencies, we relied on qualitative summary rather than pooled effect estimates. As a result, the magnitude and clinical significance of some reported changes remain uncertain.

## 7. Future Directions

To advance our understanding of how obesity interventions reshape the brain and to inform more effective treatments, future studies should consider the following: (1) Diversify cohorts and extend follow-up. Recruit participants across age ranges, ethnicities, and metabolic comorbidity profiles. Design studies with longer observation windows (years to lifelong) to capture delayed or cumulative neural effects. (2) Evaluate combined and subgroup-specific interventions. Systematically compare single-modality (diet, exercise, neuromodulation, pharmacotherapy, surgery) versus multimodal regimens. Stratify analyses by obesity phenotype (e.g., visceral vs. subcutaneous), sex, genetic risk, and coexisting metabolic disorders to identify optimal, personalized protocols. (3) Integrate multimodal neuroimaging. Combine structural MRI, task- and resting-state fMRI, DTI, electroencephalography, and molecular PET/single photon emission computed tomography to construct a multiscale atlas of obesity-related brain changes. Correlate imaging biomarkers with behavioral, metabolic, and molecular readouts for mechanistic insight. (4) Stratified analyses and subgroup meta-analyses. Pre-specify and report stratified analyses by age brackets (e.g., <40, 40–60, >60 years), sex, and key metabolic parameters. Conduct formal subgroup meta-analyses or meta-regressions to test for effect modification by these factors, which would help clarify whether the imaging biomarkers perform uniformly across clinical populations. (5) Advance precision medicine approaches. Investigate how sex, genetic polymorphisms, and comorbid conditions modulate neural plasticity following intervention. Use these insights to tailor dosing, intervention combinations, and timing, maximizing efficacy while ensuring safety and long-term compliance.

## 8. Conclusions

In summary, although the relationship between obesity and brain structure and function has been confirmed in many studies, the apparent neural mechanisms have not yet been fully elucidated; the synergistic or differentiated effects of different interventions at the cerebral neural level are still an important research topic. In the future, multimodal imaging and large-scale, long-follow-up cohort studies should be further utilized, combined with personalized intervention design, to investigate the neural substrate changes and reversibility of obesity, and to provide a more solid scientific basis and feasible clinical pathways for curbing the global obesity epidemic and improving the health of the population.

## Figures and Tables

**Table 1:** Neurocognitive and metabolic outcomes of Behavioral interventions.

Intervention Types	Target Area	Protocol	Physiological Effects	Effect Size	*p*
Lifestyle intervention	Multiple brain regions (right insula, left cingulate cortex)	18-month diet + exercise intervention	BMI reduction correlated with actual weight loss	N/A	*p* < 0.001 (Finkelstein et al., 2024) [67]
FKOWAN Aerobic exercise	Striatum, hippocampus	8 weeks	Restored cerebral insulin sensitivity, enhanced hippocampal connectivity	N/A	*p* < 0.05 (Kullmann et al., 2022) [68]
Structured exercise	Hippocampus, prefrontal cortex	20 weeks	Improved crystallized intelligence	0.72	*p* < 0.001 (Ortega et al., 2022) [31]
Combined exercise	Visceral/intermuscular fat	26 weeks	41% IMAT reduction, 36% VAT reduction	0.82	*p* < 0.05 (Waters et al., 2022) [69]
MIND diet	Cognition-related brain regions	3 months	Improved cognitive performance (FDST, BDST, LNST, SDMT, TMTA) and brain structure	working memory: 0.82	*p* < 0.05 (Arjmand et al., 2022) [70]
Mixed nuts intake	Occipital/frontal lobes	60 g daily for 16 weeks	Enhanced cerebral insulin sensitivity	N/A	*p* < 0.03 (Nijssen et al., 2023) [71]

Notes: BMI: body mass index, IMAT: intermuscular adipose tissue, VAT: visceral adipose tissue, FDST: Forward Digit Span Test, BDST: Backward Digit Span Test, LNST: Letter Number Sequencing Task, SDMT: Symbol Digit Modalities Test, TMTA: Trail Making Test Part A, MIND: Mediterranean-DASH Intervention for Neurodegenerative Delay, N/A: not mentioned.

**Table 2:** Effects of non-invasive brain stimulation on obesity.

Stimulation Types	Target Area	Protocol	Physiological Effects	Effect Size	*p*
TMS	Medial orbitofrontal cortex (mOFC)	18 Hz, 3 times/week × 5 w	Significant weight loss; increased mOFC functional connectivity	N/A	*p* < 0.001 (Devoto et al., 2021) [73]
	mOFC	18 Hz, 30 sessions, 5 weeks	Weight, BMI, waist circumference loss; metabolic improvement; gut flora changes	Weight: Cohen’s d = 0.55	*p* = 0.042 (Ferrulli et al., 2021) [75]
	Dorsolateral prefrontal cortex (DLPFC)	10 Hz (2000 pulses/trip × 4 w)	Weight loss; increased connectivity of the right frontoparietal network	Weight: Cohen’s d = 0.72	*p* < 0.001 (kim et al., 2019) [74]
tDCS	Right inferior frontal gyrus (rIFG)	2 mA anodal (20 min/day × 4 w)	Improved suppression control; RE inhibitory control deficits eliminated	Cohen’s d’ = 0.08	*p* = 0.835 (Schroeder et al., 2023) [76]
	Left DLPFC + anterior insula	2 mA (40 min/session × 15)	Reduced commission errors in Go/No-Go tasks; negative correlation with snack intake	N/A	*p* = 0.007 (Stinson et al., 2022) [78]
	L-DLPFC	2 mA, 20 sessions/4 weeks, 20 min/session	Reduces UE, EE; improves fibromyalgia symptoms	Cohen’s d’ = 0.55	*p* = 0.018 (Jornada et al., 2024) [77]
	L-DLPFC	2 mA (20 min/mission × 3 days)	Reduces IL-6 concentrations	N/A	*p* = 0.0299 (Aydin et al., 2024) [79]
	DLPFC	2 mA (20 min/session)	Inhibits reward mechanisms; improves cognitive control; reduces food cravings	0.55	*p* = 0.05 (Ghobadi-Azbari et al., 2022) [80]

Notes: RE: restricted eater, UE: uncontrolled eating, EE: emotional eating, Cohen’s d (mean difference effect size): small effect: 0.20, medium effect: 0.50, large effect: 0.80, N/A: not mentioned.

**Table 3:** Comparative efficacy of obesity pharmacotherapies.

Drug/Therapy	Mechanism of Action	Dosage Regimen	Key Effects	Sample Size	Effect Size	*p*
Liraglutide	GLP-1 receptor agonist	3.0 mg SC daily (56 weeks)	Weight loss, improved blood sugar control, lower blood pressure and lipids	3731	0.81	*p* < 0.001 (Pi-Sunyer et al., 2015) [87]
Intranasal Oxytocin	Hypothalamic reward modulation	24 IU daily	24% reduction in high-calorie snack consumption	20	0.81	*p* < 0.001 (Ott et al., 2013) [32]
		0.3/0.7 mg/kg/21 days (24 weeks)	Sustained 3.8% weight loss (AE 36% vs. placebo 31%)	120	0.69	*p* = 0.003 (Humbert et al., 2021) [88]
Central Insulin	Regulation of central insulin action	160 IU intranasal	Hunger pangs are reduced	60	N/A	*p* = 0.002 (Wagner et al., 2022) [89]
		160 IU intranasal	Amygdala BOLD signaling is enhanced	60	0.83	*p* < 0.002 (Tiedemann et al., 2022) [90]
Secretin	BAT activation + vagal signaling	20 μg IV bolus (12 weeks)	31% (±9%) ↓ high-fat meal consumption	60	N/A	*p* = 0.004 (Lee et al., 2022) [92]
GLP-1 + OT Combo	Multimodal synergy	Liraglutide + Oxytocin (52 weeks)	14.7% (±3.2%) weight loss vs. 8.9% monotherapy	350	N/A	*p* = 0.001 (Rizwan et al., 2021) [94]

Notes: BAT: brown adipose tissue, SC: subcutaneous, IV: intravenous, GLP-1: glucagon-like peptide-1, OT: oxytocin, VTA: ventral tegmental area.

## Data Availability

No new data were created or analyzed in this study.

## References

[1] Leng X., Huang Y., Zhao S., Jiang X., Shi P., Chen H. (2022). Altered neural correlates of episodic memory for food and non-food cues in females with overweight/obesity. Appetite.

[2] Koenis M.M.G., Papasavas P.K., Janssen R.J., Tishler D.S., Pearlson G.D. (2021). Brain responses to anticipatory cues and milkshake taste in obesity, and their relationship to bariatric surgery outcome. NeuroImage.

[3] Zhang P., Wu G.W., Yu F.X., Liu Y., Li M.Y., Wang Z., Ding H.Y., Li X.S., Wang H., Jin M. (2020). Abnormal Regional Neural Activity and Reorganized Neural Network in Obesity: Evidence from Resting-State fMRI. Obesity.

[4] Boutari C., Mantzoros C.S. (2022). A 2022 update on the epidemiology of obesity and a call to action: As its twin COVID-19 pandemic appears to be receding, the obesity and dysmetabolism pandemic continues to rage on. Metab. Clin. Exp..

[5] Hill J.J. (2018). Obesity: An Emerging Threat. J. Natl. Black Nurses’ Assoc. JNBNA.

[6] Xuan X., Zhang Y., Song Y., Zhang B., Liu J., Liu D., Lu S. (2024). Role of protein arginine methyltransferase 1 in obesity-related metabolic disorders: Research progress and implications. Diabetes Obes. Metab..

[7] Mao L., Lu J., Hou Y., Nie T. (2024). Directly targeting PRDM16 in thermogenic adipose tissue to treat obesity and its related metabolic diseases. Front. Endocrinol..

[8] Wang M., Min M., Duan H., Mai J., Liu X. (2024). The role of macrophage and adipocyte mitochondrial dysfunction in the pathogenesis of obesity. Front. Immunol..

[9] Li G., Hu Y., Zhang W., Wang J., Ji W., Manza P., Volkow N.D., Zhang Y., Wang G.J. (2023). Brain functional and structural magnetic resonance imaging of obesity and weight loss interventions. Mol. Psychiatry.

[10] Fuchs B.A., Pearce A.L., Rolls B.J., Wilson S.J., Rose E.J., Geier C.F., Keller K.L. (2024). Does ‘portion size’ matter? Brain responses to food and non-food cues presented in varying amounts. Appetite.

[11] Pimpini L., Kochs S., Franssen S., van den Hurk J., Valente G., Roebroeck A., Jansen A., Roefs A. (2022). More complex than you might think: Neural representations of food reward value in obesity. Appetite.

[12] Spetter M.S. (2018). Current state of the use of neuroimaging techniques to understand and alter appetite control in humans. Curr. Opin. Clin. Nutr. Metab. Care.

[13] Yeung A.W.K. (2021). Brain responses to watching food commercials compared with nonfood commercials: A meta-analysis on neuroimaging studies. Public Health Nutr..

[14] Hargrave S.L., Jones S., Davidson T.L. (2016). The Outward Spiral: A vicious cycle model of obesity and cognitive dysfunction. Curr. Opin. Behav. Sci..

[15] Hamer M., Sabia S., Batty G.D., Shipley M.J., Tabák A.G., Singh-Manoux A., Kivimaki M. (2012). Physical activity and inflammatory markers over 10 years: Follow-up in men and women from the Whitehall II cohort study. Circulation.

[16] Gold A.L., Abend R., Britton J.C., Behrens B., Farber M., Ronkin E., Chen G., Leibenluft E., Pine D.S. (2020). Age Differences in the Neural Correlates of Anxiety Disorders: An fMRI Study of Response to Learned Threat. Am. J. Psychiatry.

[17] Gautron L., Elmquist J.K., Williams K.W. (2015). Neural control of energy balance: Translating circuits to therapies. Cell.

[18] Matura S., Prvulovic D., Mohadjer N., Fusser F., Oertel V., Reif A., Pantel J., Karakaya T. (2021). Association of dietary fat composition with cognitive performance and brain morphology in cognitively healthy individuals. Acta Neuropsychiatr..

[19] Mora-Gonzalez J., Esteban-Cornejo I., Solis-Urra P., Rodriguez-Ayllon M., Cadenas-Sanchez C., Hillman C.H., Kramer A.F., Catena A., Ortega F.B. (2024). The effects of an exercise intervention on neuroelectric activity and executive function in children with overweight/obesity: The ActiveBrains randomized controlled trial. Scand. J. Med. Sci. Sports.

[20] Li L., Yu H., Zhong M., Liu S., Wei W., Meng Y., Li M.L., Li T., Wang Q. (2022). Gray matter volume alterations in subjects with overweight and obesity: Evidence from a voxel-based meta-analysis. Front. Psychiatry.

[21] Yu J., Morys F., Dagher A., Lajoie A., Gomes T., Ock E.Y., Kimoff R.J., Kaminska M. (2023). Associations between sleep-related symptoms, obesity, cardiometabolic conditions, brain structural alterations and cognition in the UK biobank. Sleep Med..

[22] Kokubun K., Pineda J.C.D., Yamakawa Y. (2021). Unhealthy lifestyles and brain condition: Examining the relations of BMI, living alone, alcohol intake, short sleep, smoking, and lack of exercise with gray matter volume. PLoS ONE.

[23] Spangaro M., Mazza E., Poletti S., Cavallaro R., Benedetti F. (2018). Obesity influences white matter integrity in schizophrenia. Psychoneuroendocrinology.

[24] Menks W.M., Furger R., Lenz C., Fehlbaum L.V., Stadler C., Raschle N.M. (2017). Microstructural White Matter Alterations in the Corpus Callosum of Girls With Conduct Disorder. J. Am. Acad. Child Adolesc. Psychiatry.

[25] Belfort-DeAguiar R., Seo D., Lacadie C., Naik S., Schmidt C., Lam W., Hwang J., Constable T., Sinha R., Sherwin R.S. (2018). Humans with obesity have disordered brain responses to food images during physiological hyperglycemia. Am. J. Physiol. Endocrinol. Metab..

[26] Avery J.A., Powell J.N., Breslin F.J., Lepping R.J., Martin L.E., Patrician T.M., Donnelly J.E., Savage C.R., Simmons W.K. (2017). Obesity is associated with altered mid-insula functional connectivity to limbic regions underlying appetitive responses to foods. J. Psychopharmacol..

[27] Ravichandran S., Bhatt R.R., Pandit B., Osadchiy V., Alaverdyan A., Vora P., Stains J., Naliboff B., Mayer E.A., Gupta A. (2021). Alterations in reward network functional connectivity are associated with increased food addiction in obese individuals. Sci. Rep..

[28] Pujol J., Blanco-Hinojo L., Martínez-Vilavella G., Deus J., Pérez-Sola V., Sunyer J. (2021). Dysfunctional Brain Reward System in Child Obesity. Cereb. Cortex.

[29] Olivo G., Wiemerslage L., Nilsson E.K., Solstrand Dahlberg L., Larsen A.L., Olaya Búcaro M., Gustafsson V.P., Titova O.E., Bandstein M., Larsson E.M. (2016). Resting-State Brain and the FTO Obesity Risk Allele: Default Mode, Sensorimotor, and Salience Network Connectivity Underlying Different Somatosensory Integration and Reward Processing between Genotypes. Front. Hum. Neurosci..

[30] Hong J., Bo T., Xi L., Xu X., He N., Zhan Y., Li W., Liang P., Chen Y., Shi J. (2021). Reversal of Functional Brain Activity Related to Gut Microbiome and Hormones After VSG Surgery in Patients With Obesity. J. Clin. Endocrinol. Metab..

[31] Ortega F.B., Mora-Gonzalez J., Cadenas-Sanchez C., Esteban-Cornejo I., Migueles J.H., Solis-Urra P., Verdejo-Román J., Rodriguez-Ayllon M., Molina-Garcia P., Ruiz J.R. (2022). Effects of an Exercise Program on Brain Health Outcomes for Children With Overweight or Obesity: The ActiveBrains Randomized Clinical Trial. JAMA Netw. Open.

[32] Ott V., Finlayson G., Lehnert H., Heitmann B., Heinrichs M., Born J., Hallschmid M. (2013). Oxytocin reduces reward-driven food intake in humans. Diabetes.

[33] Rebelos E., Latva-Rasku A., Koskensalo K., Pekkarinen L., Saukko E., Ihalainen J., Honka M.J., Tuisku J., Bucci M., Laurila S. (2024). Insulin-stimulated brain glucose uptake correlates with brain metabolites in severe obesity: A combined neuroimaging study. J. Cereb. Blood Flow Metab. Off. J. Int. Soc. Cereb. Blood Flow Metab..

[34] Htun K.T., Pan J., Pasanta D., Tungjai M., Udomtanakunchai C., Petcharoen T., Chamta N., Kosicharoen S., Chukua K., Lai C. (2021). Advanced Molecular Imaging (MRI/MRS/1H NMR) for Metabolic Information in Young Adults with Health Risk Obesity. Life.

[35] Giuliano A., Donatelli G., Cosottini M., Tosetti M., Retico A., Fantacci M.E. (2017). Hippocampal subfields at ultra high field MRI: An overview of segmentation and measurement methods. Hippocampus.

[36] Torigian D.A., Zaidi H., Kwee T.C., Saboury B., Udupa J.K., Cho Z.H., Alavi A. (2013). PET/MR imaging: Technical aspects and potential clinical applications. Radiology.

[37] Ustsinau U., Kulterer O.C., Rausch I., Krššák M., Kiefer F.W., Hacker M., Philippe C. (2024). A PET/MRI study on the effect of obesity and NAFLD on hepatic [18F]FDG uptake. Eur. J. Radiol..

[38] Korenblik V., Brouwer M.E., Korosi A., Denys D., Bockting C.L.H., Brul S., Lok A. (2023). Are neuromodulation interventions associated with changes in the gut microbiota? A systematic review. Neuropharmacology.

[39] Peeken J.C., Goldberg T., Pyka T., Bernhofer M., Wiestler B., Kessel K.A., Tafti P.D., Nüsslin F., Braun A.E., Zimmer C. (2019). Combining multimodal imaging and treatment features improves machine learning-based prognostic assessment in patients with glioblastoma multiforme. Cancer Med..

[40] Herzog N., Hartmann H., Janssen L.K., Waltmann M., Fallon S.J., Deserno L., Horstmann A. (2024). Working memory gating in obesity: Insights from a case-control fMRI study. Appetite.

[41] Meng Q., Han Y., Ji G., Li G., Hu Y., Liu L., Jin Q., von Deneen K.M., Zhao J., Cui G. (2018). Disrupted topological organization of the frontal-mesolimbic network in obese patients. Brain Imaging Behav..

[42] Kaufmann L.K., Custers E., Vreeken D., Snabel J., Morrison M.C., Kleemann R., Wiesmann M., Hazebroek E.J., Aarts E., Kiliaan A.J. (2024). Additive effects of depression and obesity on neural correlates of inhibitory control. J. Affect. Disord..

[43] Pan X., Zhang M., Tian A., Chen L., Sun Z., Wang L., Chen P. (2022). Exploring the genetic correlation between obesity-related traits and regional brain volumes: Evidence from UK Biobank cohort. NeuroImage. Clin..

[44] Liu H., Zhang X., Liu H., Chong S.T. (2023). Using Machine Learning to Predict Cognitive Impairment Among Middle-Aged and Older Chinese: A Longitudinal Study. Int. J. Public Health.

[45] Morys F., Dadar M., Dagher A. (2021). Association Between Midlife Obesity and Its Metabolic Consequences, Cerebrovascular Disease, and Cognitive Decline. J. Clin. Endocrinol. Metab..

[46] Gogniat M.A., Robinson T.L., Mewborn C.M., Jean K.R., Miller L.S. (2018). Body mass index and its relation to neuropsychological functioning and brain volume in healthy older adults. Behav. Brain Res..

[47] Pachter D., Kaplan A., Tsaban G., Zelicha H., Meir A.Y., Rinott E., Levakov G., Salti M., Yovell Y., Huhn S. (2024). Glycemic control contributes to the neuroprotective effects of Mediterranean and green-Mediterranean diets on brain age: The DIRECT PLUS brain-magnetic resonance imaging randomized controlled trial. Am. J. Clin. Nutr..

[48] Singh M.K., Leslie S.M., Packer M.M., Zaiko Y.V., Phillips O.R., Weisman E.F., Wall D.M., Jo B., Rasgon N. (2019). Brain and behavioral correlates of insulin resistance in youth with depression and obesity. Horm. Behav..

[49] Verstynen T.D., Weinstein A.M., Schneider W.W., Jakicic J.M., Rofey D.L., Erickson K.I. (2012). Increased body mass index is associated with a global and distributed decrease in white matter microstructural integrity. Psychosom. Med..

[50] Tryon M.S., Stanhope K.L., Epel E.S., Mason A.E., Brown R., Medici V., Havel P.J., Laugero K.D. (2015). Excessive Sugar Consumption May Be a Difficult Habit to Break: A View From the Brain and Body. J. Clin. Endocrinol. Metab..

[51] Almby K.E., Lundqvist M.H., Abrahamsson N., Kvernby S., Fahlström M., Pereira M.J., Gingnell M., Karlsson F.A., Fanni G., Sundbom M. (2021). Effects of Gastric Bypass Surgery on the Brain: Simultaneous Assessment of Glucose Uptake, Blood Flow, Neural Activity, and Cognitive Function During Normo- and Hypoglycemia. Diabetes.

[52] Apolzan J.W., Carmichael O.T., Kirby K.M., Ramakrishnapillai S.R., Beyl R.A., Martin C.K. (2021). The effects of the form of sugar (solid vs. beverage) on body weight and fMRI activation: A randomized controlled pilot study. PLoS ONE.

[53] Edwin Thanarajah S., DiFeliceantonio A.G., Albus K., Kuzmanovic B., Rigoux L., Iglesias S., Hanßen R., Schlamann M., Cornely O.A., Brüning J.C. (2023). Habitual daily intake of a sweet and fatty snack modulates reward processing in humans. Cell Metab..

[54] Farr O.M. (2021). Acute diet soda consumption alters brain responses to food cues in humans: A randomized, controlled, cross-over pilot study. Nutr. Health.

[55] van Bloemendaal L., Veltman D.J., ten Kulve J.S., Drent M.L., Barkhof F., Diamant M., IJzerman R.G. (2015). Emotional eating is associated with increased brain responses to food-cues and reduced sensitivity to GLP-1 receptor activation. Obesity.

[56] de Groot C., Felius A., Trompet S., de Craen A.J., Blauw G.J., van Buchem M.A., Delemarre-van de Waal H.A., van der Grond J. (2015). Association of the fat mass and obesity-associated gene risk allele, rs9939609A, and reward-related brain structures. Obesity.

[57] Eriksson J.W., Pereira M.J., Kagios C., Kvernby S., Lundström E., Fanni G., Lundqvist M.H., Carlsson B.C.L., Sundbom M., Tarai S. (2024). Short-term effects of obesity surgery versus low-energy diet on body composition and tissue-specific glucose uptake: A randomised clinical study using whole-body integrated 18F-FDG-PET/MRI. Diabetologia.

[58] Legget K.T., Cornier M.A., Erpelding C., Lawful B.P., Bear J.J., Kronberg E., Tregellas J.R. (2022). An implicit priming intervention alters brain and behavioral responses to high-calorie foods: A randomized controlled study. Am. J. Clin. Nutr..

[59] Mehlhose C., Risius A. (2020). Signs of Warning: Do Health Warning Messages on Sweets Affect the Neural Prefrontal Cortex Activity?. Nutrients.

[60] Dodd S.L., Long J.D., Hou J., Kahathuduwa C.N., O′Boyle M.W. (2020). Brain activation and affective judgements in response to personal dietary images: An fMRI preliminary study. Appetite.

[61] Rotenstein L.S., Sheridan M., Garg R., Adler G.K. (2015). Effect of mineralocorticoid receptor blockade on hippocampal-dependent memory in adults with obesity. Obesity.

[62] Hanssen R., Rigoux L., Kuzmanovic B., Iglesias S., Kretschmer A.C., Schlamann M., Albus K., Edwin Thanarajah S., Sitnikow T., Melzer C. (2023). Liraglutide restores impaired associative learning in individuals with obesity. Nat. Metab..

[63] Anguah K.O.B., Christ S.E. (2024). Exposure to written content eliciting weight stigmatization: Neural responses in appetitive and food reward regions. Obesity.

[64] Chumachenko S.Y., Cali R.J., Rosal M.C., Allison J.J., Person S.J., Ziedonis D., Nephew B.C., Moore C.M., Zhang N., King J.A. (2021). Keeping weight off: Mindfulness-Based Stress Reduction alters amygdala functional connectivity during weight loss maintenance in a randomized control trial. PLoS ONE.

[65] Alessi J., Dzemidzic M., Harezlak J., Kareken D.A., Considine R.V. (2024). Neural processing of sweet taste in reward regions is reduced following bariatric surgery. Obesity.

[66] Wingrove J.O., O′Daly O., Forbes B., Swedrowska M., Amiel S.A., Zelaya F.O. (2021). Intranasal insulin administration decreases cerebral blood flow in cortico-limbic regions: A neuropharmacological imaging study in normal and overweight males. Diabetes Obes. Metab..

[67] Finkelstein O., Levakov G., Kaplan A., Zelicha H., Meir A.Y., Rinott E., Tsaban G., Witte A.V., Blüher M., Stumvoll M. (2024). Deep learning-based BMI inference from structural brain MRI reflects brain alterations following lifestyle intervention. Hum. Brain Mapp..

[68] Kullmann S., Goj T., Veit R., Fritsche L., Wagner L., Schneeweiss P., Hoene M., Hoffmann C., Machann J., Niess A. (2022). Exercise restores brain insulin sensitivity in sedentary adults who are overweight and obese. JCI Insight.

[69] Waters D.L., Aguirre L., Gurney B., Sinacore D.R., Fowler K., Gregori G., Armamento-Villareal R., Qualls C., Villareal D.T. (2022). Effect of Aerobic or Resistance Exercise, or Both, on Intermuscular and Visceral Fat and Physical and Metabolic Function in Older Adults With Obesity While Dieting. J. Gerontol. Ser. A Biol. Sci. Med. Sci..

[70] Arjmand G., Abbas-Zadeh M., Eftekhari M.H. (2022). Effect of MIND diet intervention on cognitive performance and brain structure in healthy obese women: A randomized controlled trial. Sci. Rep..

[71] Nijssen K.M., Mensink R.P., Plat J., Ivanov D., Preissl H., Joris P.J. (2024). Mixed nut consumption improves brain insulin sensitivity: A randomized, single-blinded, controlled, crossover trial in older adults with overweight or obesity. Am. J. Clin. Nutr..

[72] Stice E., Yokum S., Gau J., Veling H., Lawrence N., Kemps E. (2022). Efficacy of a food response and attention training treatment for obesity: A randomized placebo controlled trial. Behav. Res. Ther..

[73] Devoto F., Ferrulli A., Zapparoli L., Massarini S., Banfi G., Paulesu E., Luzi L. (2021). Repetitive deep TMS for the reduction of body weight: Bimodal effect on the functional brain connectivity in “diabesity”. Nutr. Metab. Cardiovasc. Dis. NMCD.

[74] Kim S.H., Park B.Y., Byeon K., Park H., Kim Y., Eun Y.M., Chung J.H. (2019). The effects of high-frequency repetitive transcranial magnetic stimulation on resting-state functional connectivity in obese adults. Diabetes Obes. Metab..

[75] Ferrulli A., Drago L., Gandini S., Massarini S., Bellerba F., Senesi P., Terruzzi I., Luzi L. (2021). Deep Transcranial Magnetic Stimulation Affects Gut Microbiota Composition in Obesity: Results of Randomized Clinical Trial. Int. J. Mol. Sci..

[76] Schroeder P.A., Farshad M., Svaldi J. (2023). Anodal stimulation of inhibitory control and craving in satiated restrained eaters. Nutr. Neurosci..

[77] Jornada M.N.D., Antunes L.C., Alves C., Torres I.L.S., Fregni F., S Sanches P.R., P Silva D., Caumo W. (2024). Impact of multiple-session home-based transcranial direct current stimulation (M-HB-tDCS) on eating behavior in fibromyalgia: A factorial randomized clinical trial. Brain Stimul..

[78] Stinson E.J., Travis K.T., Magerowski G., Alonso-Alonso M., Krakoff J., Gluck M.E. (2022). Improved food Go/No-Go scores after transcranial direct current stimulation (tDCS) to prefrontal cortex in a randomized trial. Obesity.

[79] Aydin B.N., Stinson E.J., Travis K.T., Krakoff J., Rodzevik T., Chang D.C., Gluck M.E. (2024). Reduced plasma interleukin-6 concentration after transcranial direct current stimulation to the prefrontal cortex. Behav. Brain Res..

[80] Ghobadi-Azbari P., Jamil A., Yavari F., Esmaeilpour Z., Malmir N., Mahdavifar-Khayati R., Soleimani G., Cha Y.H., Shereen A.D., Nitsche M.A. (2021). fMRI and transcranial electrical stimulation (tES): A systematic review of parameter space and outcomes. Prog. Neuro-Psychopharmacol. Biol. Psychiatry.

[81] Strafella A.P., Paus T., Barrett J., Dagher A. (2001). Repetitive transcranial magnetic stimulation of the human prefrontal cortex induces dopamine release in the caudate nucleus. J. Neurosci..

[82] McKlveen J.M., Myers B., Herman J.P. (2015). The medial prefrontal cortex: Coordinator of autonomic, neuroendocrine and behavioural responses to stress. J. Neuroendocrinol..

[83] Stagg C.J., Nitsche M.A. (2011). Physiological basis of transcranial direct current stimulation. Neuroscientist.

[84] Wagner J., Makeig S., Hoopes D., Gola M. (2019). Can oscillatory alpha-gamma phase-amplitude coupling be used to understand and enhance TMS effects?. Front. Hum. Neurosci..

[85] Xiao J., Wang T., Xu Y., Gu X., Li D., Niu K., Wang T., Zhao J., Zhou R., Wang H.L. (2020). Long-term probiotic intervention mitigates memory dysfunction through a novel H3K27me3-based mechanism in lead-exposed rats. Transl. Psychiatry.

[86] Breit S., Kupferberg A., Rogler G., Hasler G. (2018). Vagus nerve as modulator of the brain-gut axis in psychiatric and inflammatory disorders. Front. Psychiatry.

[87] Pi-Sunyer X., Astrup A., Fujioka K., Greenway F., Halpern A., Krempf M., Lau D.C., le Roux C.W., Violante Ortiz R., Jensen C.B. (2015). SCALE Obesity and Prediabetes NN8022-1839 Study Group A Randomized, Controlled Trial of 3.0 mg of Liraglutide in Weight Management. N. Engl. J. Med..

[88] Humbert M., McLaughlin V., Gibbs J.S.R., Gomberg-Maitland M., Hoeper M.M., Preston I.R., Souza R., Waxman A., Escribano Subias P., Feldman J. (2021). Sotatercept for the Treatment of Pulmonary Arterial Hypertension. N. Engl. J. Med..

[89] Wagner L., Veit R., Fritsche L., Häring H.U., Fritsche A., Birkenfeld A.L., Heni M., Preissl H., Kullmann S. (2022). Sex differences in central insulin action: Effect of intranasal insulin on neural food cue reactivity in adults with normal weight and overweight. Int. J. Obes..

[90] Tiedemann L.J., Meyhöfer S.M., Francke P., Beck J., Büchel C., Brassen S. (2022). Insulin sensitivity in mesolimbic pathways predicts and improves with weight loss in older dieters. Elife.

[91] Horne B.D., Anderson J.L., May H.T., Bair T.L., Le V.T., Iverson L., Knowlton K.U., Muhlestein J.B. (2024). Insulin resistance reduction, intermittent fasting, and human growth hormone: Secondary analysis of a randomized trial. Npj Metab. Health Dis..

[92] Lee H.J., Jin B.Y., Park M.R., Kim N.H., Seo K.S., Jeong Y.T., Wada T., Lee J.S., Choi S.H., Kim D.H. (2023). Inhibition of adipose tissue angiogenesis prevents rebound weight gain after caloric restriction in mice fed a high-fat diet. Life Sci..

[93] Hinte L.C., Castellano-Castillo D., Ghosh A., Melrose K., Gasser E., Noé F., Massier L., Dong H., Sun W., Hoffmann A. (2024). Adipose tissue retains an epigenetic memory of obesity after weight loss. Nature.

[94] Rizwan M., Aslam N., Ashfaq U.A., Hayat M., Hussain S.M. (2021). SNP of HMGCR and Apo E genes and their impact in response to statin therapy in hypercholesterolemic and hypertriglyceridemic patients in Pakistan. Pak. J. Pharm. Sci..

